# Transition in eye gaze as a predictor of emergence from general anesthesia in children and adults: a prospective observational study

**DOI:** 10.1186/s12871-022-01867-3

**Published:** 2022-10-17

**Authors:** Michiko Kinoshita, Yoko Sakai, Kimiko Katome, Tomomi Matsumoto, Shizuka Sakurai, Yuka Jinnouchi, Katsuya Tanaka

**Affiliations:** Department of Anesthesiology, Tokushima University Hospital, 2-50-1 Kuramoto-cho, Tokushima-shi, Tokushima, 770-8503 Japan

**Keywords:** Eye gaze, Emergence, General anesthesia

## Abstract

**Background:**

It is useful to monitor eye movements during general anesthesia, but few studies have examined neurological finding of the eyes during emergence from general anesthesia maintained with short-acting opioids and volatile anesthetics.

**Methods:**

Thirty children aged 1–6 years and 30 adults aged 20–79 years were enrolled. Patients received general anesthesia maintained with sevoflurane and remifentanil. The timing of three physical-behavioral responses—eye-gaze transition (the cycle from conjugate to disconjugate and back to conjugate), resumption of somatic movement (limbs or body), and resumption of respiration—were recorded until spontaneous awakening. The primary outcome measure was the timing of the physical-behavioral responses. Secondary outcome measures were the incidence of eye-gaze transition, and the bispectral index, concentration of end-tidal sevoflurane, and heart rate at the timing of eye-gaze transition.

**Results:**

Eye-gaze transition was evident in 29 children (96.7%; 95% confidence interval, 82.8–99.9). After the end of surgery, eye-gaze transition was observed significantly earlier than resumption of somatic movement or respiration (472 [standard deviation 219] s, 723 [235] s, and 754 [232] s, respectively; *p* < 0.001). In adults, 3 cases (10%; 95% CI, 0.2–26.5) showed eye-gaze transition during emergence from anesthesia. The incidence of eye-gaze transition was significantly lower in adults than in children (*p* < 0.001).

**Conclusion:**

In children, eye-gaze transition was observed significantly earlier than other physical-behavioral responses during emergence from general anesthesia and seemed to reflect emergence from anesthesia. In contrast, observation of eye gaze was not a useful indicator of emergence from anesthesia in adults.

**Supplementary Information:**

The online version contains supplementary material available at 10.1186/s12871-022-01867-3.

## Background

Despite the development of electroencephalographic monitoring, neurologic examinations can provide anesthesiologists with a great deal of information [1]. Because the eyes are under the control of cranial nerves, eye movements and brainstem function are closely related. Pupil dilation and the position of the eyes have long been observed as indicators of anesthetic depth [2]. The positions of the eyes show a slight divergence and elevation during deep anesthesia [3, 4], and eye movements become conjugate during wakefulness. Disconjugate gaze is known to indicate brainstem problems in non-anesthetized patients [5, 6] as well as shallow anesthesia (stage 2 of Guedel’s classification [7, 8]) in anesthetized patients. However, because Guedel’s classification is based on anesthesia induced with inhalation anesthetics alone, this classification is not applicable to the current mainstream general anesthesia techniques maintained with multiple agents. Although it is useful to observe eye movements during general anesthesia, few studies have examined the transition of eye gaze during emergence from general anesthesia maintained with short-acting opioids and volatile anesthetics.

The purpose of this study was to investigate how eye positions change in children and adults during emergence from general anesthesia maintained with remifentanil and sevoflurane. We hypothesized that changes in eye positions would appear earlier than other physical responses in both children and adults. We examined the timing of three physical-behavioral responses during emergence: eye-gaze transition, resumption of somatic movement (limbs or body), and resumption of respiration. We also investigated the incidence of eye-gaze transition and the bispectral index (BIS), concentration of end-tidal sevoflurane, and heart rate (HR) at the timing of eye-gaze transition between the end of surgery and extubation. Considering that observation of eye gaze is noninvasive and simple, and requires no training, the results of this study suggest additional procedures that can be immediately implemented in clinical practice.

## Methods

This study was approved by the Ethics Committee of Tokushima University Hospital (approval nos. 3528, 3596). Written informed consent was obtained from all participants or their parents. This manuscript adheres to the Strengthening the Reporting of Observational Studies in Epidemiology (STROBE) statement.

The eligibility criteria were as follows: scheduled for surgery and general anesthesia; evaluations performed in advance at our hospital’s outpatient anesthesiology department; American Society of Anesthesiologists physical status of I–II, which is assumed to be a stable general condition; and age 1–6 years (children) or 20–79 years (adults). Exclusion criteria were as follows: ophthalmic surgery, neurosurgery, plastic surgery above the neck, and surgery on the airway; surgery in the lateral or prone position; history of neuromuscular, ocular, or mental disease; and scheduled for postoperative sedation. Patients whose surgery could not be completed between 8:30 AM and 6:00 PM were also excluded.

Volatile induction of anesthesia with sevoflurane and nitrous oxide/oxygen was performed in children. Rocuronium was administered prior to intubation. Nitrous oxide was terminated after intubation, and an oxygen/air mixture was used for maintenance. Rapid sequence induction with propofol, remifentanil, and rocuronium was performed in adults. Both children and adults received sevoflurane (1%–3%), along with remifentanil (0.05–0.5 μg/kg/min) to maintain anesthesia and rocuronium to maintain paralysis. The doses of anesthetic agents used for maintenance were adjusted by the anesthesiologists in charge according to their usual practice at our institution, which was within the proper range approved in Japan. No particular settings were specified for this observational study. Acetaminophen, nonsteroidal anti-inflammatory drugs, regional anesthesia (e.g., peripheral nerve blocks and epidural anesthesia), and opioids (e.g., fentanyl) were used alone or in combination prior to the end of surgery for appropriate postoperative analgesia. At the end of the operation, continuous administration of sevoflurane and remifentanil was terminated simultaneously. Sugammadex was administered immediately as needed. No unnecessary stimulations followed termination of continuous anesthesia, and we waited for the patient to awaken spontaneously. Mechanical ventilation was maintained by keeping end-tidal CO_2_ at 35–45 mmHg until somatic movement or respiration was observed. After awakening, airway suctioning was performed before or after extubation as needed.

Eye gaze was monitored at least every 15 s after the end of the operation. Gaze was checked visually by gently opening the eyelids. Vital signs routinely monitored during anesthesia were also recorded. The times from termination of continuous anesthetic administration at the end of surgery to the initial onset of somatic movement and respiration were recorded in units of seconds. Extubation was performed in children when conjugate eye gaze, somatic movement, and spontaneous respiration were confirmed. For adults, the ability to respond to commands was an additional criterion for extubation. The presence of complications related to the inadequate awakening, including hypoxia and unconsciousness, were also recorded after extubation.

**Fig. 1:**
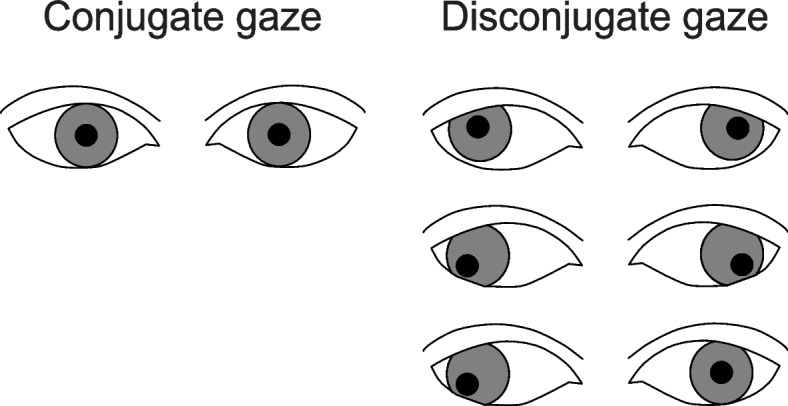
Representative images of eye-gaze transition

The primary outcome measure was the timing of the three physical-behavioral responses: eye-gaze transition (in the cycle from conjugate to disconjugate and back to conjugate; Fig. 1), resumption of somatic movement, and resumption of respiration. The secondary outcome measures were the incidence of this eye-gaze transition, and BIS, concentration of end-tidal sevoflurane, and HR at the timing of eye-gaze transition between the end of surgery and extubation. Age-adjusted minimum alveolar concentration (MAC_age_) and the end-tidal MAC_age_ fraction were calculated as follows [9].

$${\text{MAC}}_{\text{age}}={\text{MAC}}_{40} \times {10}^{-0.00269({\text{age}}-40)}$$

$$\text{End-tidal }{\text{MAC}}_{\text{age}} \text{fraction }= \, \frac{\text{End-tidal sevoflurane concentration}}{{\text{MAC}}_{\text{age}}}$$

For statistical analysis, the sample size was calculated as follows in order to compare the timing of eye-gaze transition and resumption of somatic movement and respiration with repeated-measures analysis of variance (ANOVA). The effect size f was set to a medium value of 0.25. When the α error was set to 0.05 and the power to 0.8, the sample size was calculated to be 28. All calculations were performed using G*Power ver. 3.1.9.4 (Heinrich Heine University, Düsseldorf, Germany). Rounding up, we decided on a sample size of 30 for both children and adults.

Data are shown as means (standard deviation or 95% confidence interval [95% CI]). Multi-group comparisons and post-hoc tests of repeated measures were performed by repeated-measures ANOVA followed by a paired *t*-test (Bonferroni correction). Ratios were compared by the chi-square test or Fisher’s exact test if there were five or fewer cells. All *p*-values were two-sided, and those less than 0.05 were considered statistically significant. Statistical analyses were performed using EZR (Saitama Medical Center, Jichi Medical University, Saitama, Japan), which is a graphical user interface for R version 3.6.1 (The R Foundation for Statistical Computing, Vienna, Austria) that provides statistical functions frequently used in biostatistics [10].

## Results

**Fig. 2:**
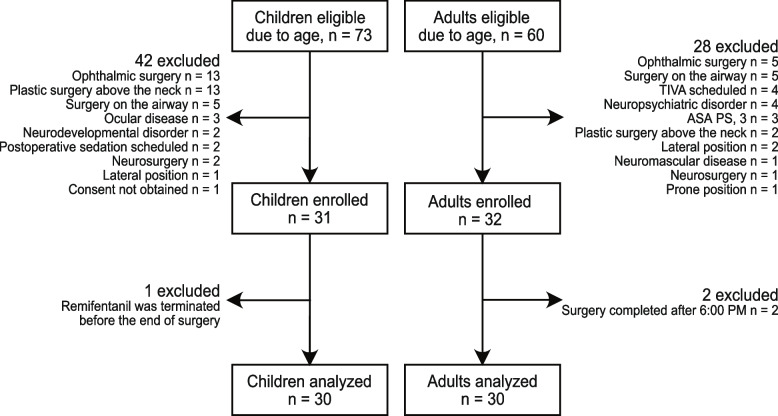
Study profile. ENT, ear, nose, and throat; TIVA, total intravenous anesthesia; ASA PS, American Society of Anesthesiologists physical status

After the study was approved by the Ethics Committee, patients who had previously been evaluated at our hospital’s outpatient department of anesthesiology between September and December 2019 were enrolled consecutively until the sample size reached 30 for both children and adults. A total of 73 children were enrolled according to age, but 42 were excluded based on pre-set exclusion criteria and 1 was excluded because the administration of remifentanil was terminated before the end of the operation. A total of 60 adults were enrolled according to age, but 28 were excluded based on pre-set exclusion criteria and 2 were excluded because their surgery continued past 06:00 PM. Figure 2 shows a flowchart of the recruitment process. The patient characteristics are shown in Table 1.

**Table 1:** Background of the patients

	Children (*n* = 30)	Adults (*n* = 30)
Age (months in children, years in adults)	27 (18–42); range 12–79	52 (44–67); range 33–79
Body weight (kg)	12.0 (10.5–14.0); range 8.2–20.0	60.0 (52.4–67.5); range 41.0–86.2
Male [% (n)]	60% (18)	27% (8)
Concentration of anesthetics at the end of surgery		
End-tidal sevoflurane concentration (%)	1.95 (1.60–2.10); range 1.25–2.21	0.95 (0.70–1.10); range 0.60–1.36
End-tidal MAC_age_ fraction	0.86 (0.70–0.92); range 0.55–0.98	0.55 (0.44–0.67); range 0.36–0.78
Remifentanil (μg ∙ kg^−1^ ∙ min^−1^)	0.20 (0.15–0.24); range 0.05–0.30	0.10 (0.10–0.15); range 0.05–0.18
Type of surgery		
Gastrointestinal surgery [% (n)]	0% (0)	17% (5)
Abdominoplasty [% (n)]	63% (19)	7% (2)
ENT surgery [% (n)]	7% (2)	7% (2)
Plastic surgery [% (n)]	7% (2)	3% (1)
Urologic surgery [% (n)]	23% (7)	10% (3)
Breast surgery [% (n)]	0% (0)	17% (5)
Gynecological surgery [% (n)]	0% (0)	40% (12)
Duration of surgical procedure (min)	52 (40–65); range 23–165	114 (76–176); range 29–247
Duration of anesthesia (min)	101 (81–116); range 69–204	160 (122–219); range 62–297

Data are presented as medians with interquartile range and the range, unless otherwise stated.

*N* Number, *MAC*_*age*_, Age-adjusted minimum alveolar concentration, *ENT* Ear, nose, and throat

**Fig. 3:**
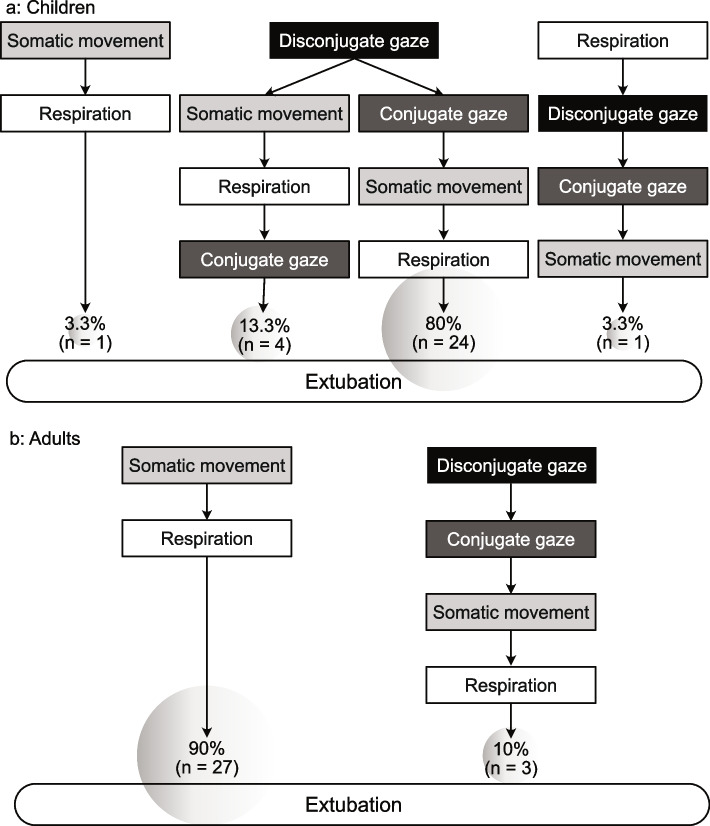
Order of onset for physical-behavioral findings after the end of the operation (**a**) Children. (**b**) Adults

Figure 3 shows the observed orders of onset for the three physical-behavioral responses (eye-gaze transition, somatic movement, and respiration) until extubation. Twenty-nine (96.7%; 95% CI, 82.8–99.9) of the 30 children showed eye-gaze transition in the cycle from conjugate to disconjugate to conjugate during emergence from general anesthesia. The earliest responses observed were transition to disconjugate gaze in 28 cases, resumption of somatic movement in 1 case, and resumption of respiration in 1 case. Three (10%; 95% CI, 0.2–26.5) of the 30 adults showed eye-gaze transition. The adults had a significantly lower incidence of eye-gaze transition compared with the children (*p* < 0.001). The earliest responses observed were transition to disconjugate gaze in 3 cases and somatic movement in 27 cases.

In children, time from the end of administration of anesthetics until the shift to disconjugate gaze, resumption of somatic movement, and resumption of respiration was 472 (219) s, 723 (235) s, and 754 (232) s, respectively. Repeated-measures ANOVA detected significant differences among these findings (*p* < 0.001). A paired *t*-test as a post-hoc comparison (Bonferroni correction) revealed that the shift to disconjugate gaze was observed significantly earlier than resumption of somatic movement (mean difference, 251 [95% CI, 178–324] s, *p* < 0.001) or respiration (mean difference, 287 [95% CI, 187–387] s, *p* < 0.001).

**Fig. 4:**
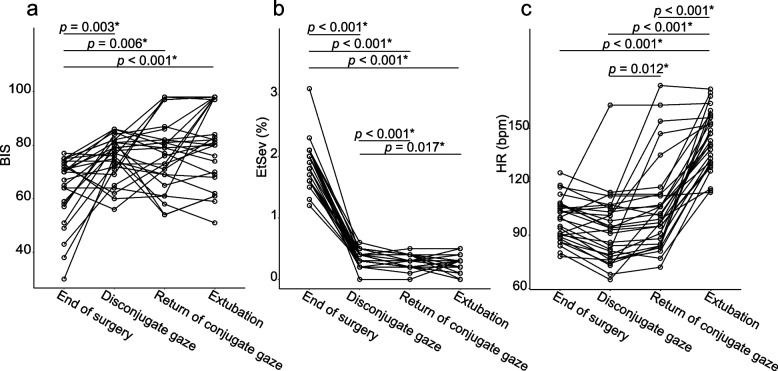
Bispectral index (BIS), end-tidal sevoflurane concentration (EtSev), and heart rate (HR) at the timing of eye-gaze transition between the end of surgery and extubation in children. **a** Individual BIS value. **b** Individual end-tidal sevoflurane concentration. **c** Individual HR. *statistically significant nLine graphs represent repeated measurement data; each point represents an individual measurement

Figure 4a shows the change in BIS at the time of the eye-gaze transition in children. BIS at the end of surgery was significantly lower than at the shift to disconjugate gaze, at the return of conjugate gaze, and at extubation (mean difference vs. disconjugate gaze, 11 [95% CI, 5.4–16.8], *p* = 0.003; vs. the return of conjugate gaze, 12 [95% CI, 5.5–18.7], *p* = 0.006; vs. extubation, 16 [95% CI, 9.1–23.4], *p* < 0.001, paired *t*-test [Bonferroni correction] after repeated measure of ANOVA). There were no differences in BIS from the shift to disconjugate gaze until extubation.

Figure 4b shows the concentration of end-tidal sevoflurane at the time of the eye-gaze transition in children. The concentration at the end of surgery was significantly higher than at the shift to disconjugate gaze, at the return of conjugate gaze, and at extubation (mean difference vs. disconjugate gaze, 1.5% [95% CI, 1.4–1.7] , *p* < 0.001; vs. the return of conjugate gaze, 1.6% [95% CI, 1.5–1.7], *p* < 0.001; vs. extubation, 1.6% [95% CI, 1.5–1.8], *p* < 0.001, paired *t*-test [Bonferroni correction] after repeated-measures ANOVA). The concentration of sevoflurane at the shift to disconjugate gaze was significantly higher than at the return of conjugate gaze and at extubation, but the mean difference was clinically small (mean difference vs. re-conjugate gaze, 0.07% [95% CI, 0.04–0.10], *p* < 0.001; vs. extubation, 0.09% [95% CI, 0.03–0.15], *p* = 0.017, paired *t*-test [Bonferroni correction]). There were no differences in the concentration between the return of conjugate gaze and at extubation. The data of Fig. 4b were converted to the end-tidal MAC_age_ fraction, and the results are shown in Additional file 1.

Figure 4c shows the change in HR at the time of the eye-gaze transition in children. HR at extubation was significantly higher than that at the end of surgery, at the shift to disconjugate gaze, and at the return of conjugate gaze (mean difference vs. end of surgery, 45 [95% CI, 37–52] beats per minute [bpm], *p* < 0.001; vs. disconjugate gaze, 50 [95% CI, 41–58] bpm, *p* < 0.001; vs. return of conjugate gaze, 37 [95% CI, 28–46] bpm, *p* < 0.001, paired *t*-test [Bonferroni correction] after repeated measure of ANOVA). There was a significant difference in HR between the shift to disconjugate gaze and the return of conjugate gaze (mean difference, 13 [95% CI, 5–20] bpm, *p* = 0.012, paired *t*-test [Bonferroni correction]).

Because the incidence of eye-gaze transition was low in adults (*n* = 3), we could not analyze BIS, concentration of end-tidal sevoflurane, and HR at the timing of eye-gaze transition. There were no cases with problems related to inadequate awakening after extubation.

## Discussion

The eye-gaze transition from conjugate to disconjugate to conjugate was frequently observed in children during emergence from general anesthesia maintained with sevoflurane and remifentanil, and this transition was observed significantly earlier than the other physical-behavioral responses, namely, resumption of somatic movement and respiration. This is contrary to the conventional view that respiration is the first physical response to recover during emergence from general anesthesia [1, 11]. Withdrawal from anesthesia maintained with remifentanil and sevoflurane resulted in delayed resumption of respiration.

BIS increased at the shift to disconjugate gaze but changed less after that, at the return of conjugate gaze and at extubation. The range of BIS values varied greatly between individuals. This finding probably means that BIS does not track the transition from shallow anesthesia to awakening well in children. Previous studies have suggested that BIS has limited utility for evaluating anesthetic depth in children [12–15]. The concentration of end-tidal sevoflurane decreased substantially at the shift to disconjugate gaze and then decreased only slightly at the return of conjugate gaze. Our results support the finding of a previous study that low concentrations of end-tidal sevoflurane do not adequately reflect emergence from anesthesia in children [16]. HR did not change significantly at the shift to disconjugate gaze but did increase at the return of conjugate gaze before increasing substantially at extubation. Findings based on these monitors seem insufficient to determine the recovery process from general anesthesia in children. The present results suggest that neurological finding of the eyes may help supplement this deficiency.

Compared with children, eye-gaze transitions occurred less frequently in adults. This suggests that eye-position information is less useful for observing emergence from general anesthesia in adults. Although the present study design cannot clarify why there is a difference in the incidence of eye-gaze transition between children and adults in, several possibilities can be considered. Adults and children require different amounts of anesthetics [9, 17, 18], which may have affected the outcome. Although anesthetic use naturally differed between the children and adults, the children received a higher dose of sevoflurane even after MAC_age_ conversion in this study. Different concentrations of anesthetics used for maintenance may affect the quality of emergence from general anesthesia; Higher concentrations of sevoflurane are reported to be associated with postoperative agitation [19]. It is also possible that the different methods for induction of anesthesia in adults and children may have influenced the results. In this study, propofol was used to induce anesthesia in adults but not in children. After intravenous bolus administration of 2 mg/kg propofol in the average adult model of this study (a 60-year-old woman weighing 60 kg), the simulated effect site concentrations at 30, 60, and 120 min were 0.35, 0.15, and 0.05 μg/ml, respectively [20]. Because the mean operative and anesthesia times in the adult group were 114 min and 160 min, respectively, the effect site concentrations of propofol were assumed to be very small at the period of emergence. However, we cannot rule out the possibility that differences in the method used for induction may have affected the results. Differences in the development of the central nervous system between children and adults might also have influenced the outcome. Age-related morphological and functional changes in the brain are known to alter electroencephalography (EEG) [21, 22], and aging decreases EEG amplitude during wakefulness, sleep, and general anesthesia [23–25]. Sevoflurane-based anesthesia shifts EEG readings toward higher frequencies with age [26]. Alpha coherence, an EEG feature related to sevoflurane-induced unconsciousness, has also been reported to vary significantly with age [27, 28]. Moreover, spectral EEG pattern characteristics during emergence from sevoflurane anesthesia have been reported to differ with age [29, 30]. Because this study used BIS but did not examine detailed EEG components, the changes in EEG during emergence from general anesthesia could not be determined. More detailed results might have been obtained if EEG had been performed instead.

This study has the following limitations. First, it examined cases in which general anesthesia was maintained with remifentanil and sevoflurane, so the results may not be generalizable to other types of anesthesia such as total intravenous anesthesia. Second, because the blood concentration of remifentanil was not measured, the effect of remifentanil during emergence from anesthesia is not known. Third, we did not stimulate the patients to wake them up because we wanted to observe their spontaneous emergence from anesthesia. Accordingly, the results of this study may not be applicable to patients who are stimulated to wake them up. The eyelids were manually opened as gently as possible to check the gaze, and it is possible that this had some effect on the patient’s awakening. Fourth, this study had a small population size. Results from a larger population would be of greater clinical importance. Finally, there was a large age gap between the children and adult groups in this study. This study could not explain the change in eye-gaze transition in the growth process. Accordingly, the results of this study cannot be applied to adolescents or young adults.

During emergence from general anesthesia maintained with sevoflurane and remifentanil, eye-gaze transition was observed frequently in children but not in adults. In addition, eye-gaze transition was observed significantly earlier compared with other findings. Observation of eye gaze is recommended as a non-invasive and simple method to infer the depth of anesthesia during emergence from anesthesia in children.

## Supplementary Information

**Additional file 1 .**

## Data Availability

The datasets used and/or analyzed during the study are available from the corresponding author on reasonable request.

## Abbreviations

ANOVA: Analysis of variance
BIS: Bispectral index
CI: Confidence interval
HR: Heart rate
MAC_age_: Age-adjusted minimum alveolar concentration
