# Supplementary figures and images for: Transition in eye gaze as a predictor of emergence from general anesthesia in children and adults: a prospective observational study
Source: BMC Anesthesiol. 2022 Oct 17;22:320. doi: 10.1186/s12871-022-01867-3 (PMC9575208; doi:10.1186/s12871-022-01867-3)

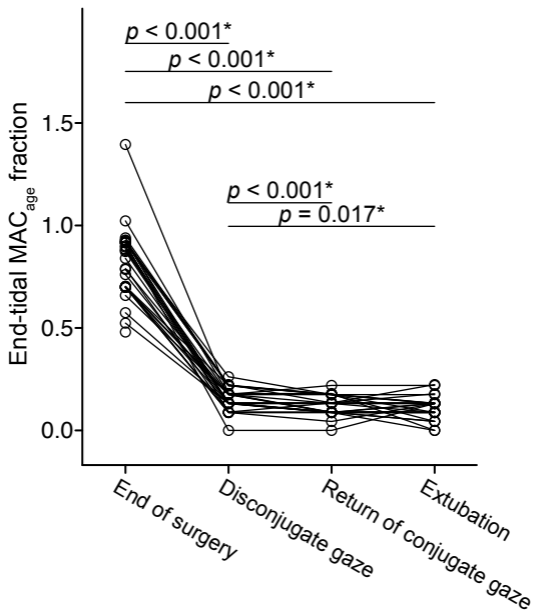

Supplement: Supplementary file 1 — Additional file 1 . [file 12871_2022_1867_MOESM1_ESM.pdf]
